# Atypical Fibroxanthoma With Osteoclast-Like Giant Cells: A Report of a Rare Histopathological Entity and Review of the Literature

**DOI:** 10.7759/cureus.117798

**Published:** 2026-10-12

**Authors:** Andrew B Martinelli, Barry White, Philip R Cohen

**Affiliations:** 1 Science Department, Highland Park Independent School District, Dallas, USA; 2 Dermatologic Surgery Department, Texas Skin Surgery Center, Plano, USA; 3 Dermatopathology Department, Sagis Dermatopathology, Houston, USA; 4 Dermatology Department, University of California Davis Health, Sacramento, USA; 5 Dermatology Department, Touro University California College of Osteopathic Medicine, Vallejo, USA

**Keywords:** atypical, cancer, cell, excision, fibroxanthoma, giant, malignancy, mohs surgery, osteoclast-like, sarcoma

## Abstract

Atypical fibroxanthoma, a malignant fibrohistiocytic tumor in the dermis, is considered to be a superficial version of the more aggressive pleomorphic dermal sarcoma. It occurs primarily in sun-exposed areas, particularly the head and neck region of elderly individuals. Surgical excision, especially with Mohs micrographic surgery, is generally curative. Atypical fibroxanthoma may exhibit a range of histopathologic features. It classically demonstrates pleomorphic cells arranged in a spindle pattern; in addition, atypical epithelioid cells with mitoses are commonly present. Additional microscopic features may also include granular areas, hemorrhage, keloid-like areas, myxoid change, and pseudoangiomatous features. Atypical fibroxanthoma with osteoclast-like giant cells is an exceedingly rare entity. In this paper, we present a 79-year-old Caucasian woman who developed a rapidly growing ulcerated nodule on the right lateral eyebrow and adjacent right upper eyelid. A shave biopsy revealed a malignant dermal tumor with not only numerous osteoclast-like giant cells but also atypical spindle cells with hyperchromatic nuclei and occasional mitotic figures in the background. Immunohistochemical stains were performed and demonstrated positive staining of the tumor cells for cluster of differentiation 10 (CD10) and cluster of differentiation 68 (CD68); the osteoclast-like giant cells also showed positive staining for CD68. The tumor cells did not stain with S100, SRY-related HMG-box 10 (SOX-10), Preferentially Expressed Antigen in Melanoma (PRAME), cytokeratin AE1/AE3, and cytokeratin 5/6 stains. Correlation of the clinical presentation, histopathology, and immunohistochemical findings established the diagnosis of an atypical fibroxanthoma with osteoclast-like giant cells. The tumor was excised via the Mohs micrographic technique, and clear margins were obtained after the first stage; the resulting defect extended to superficial muscular fascia and was repaired with a local advancement flap. We report a patient with a rare variant of atypical fibroxanthoma presenting with osteoclast-like giant cells and review the literature regarding this uncommon entity.

## Introduction

Atypical fibroxanthoma is a primary, dermal-based malignancy that occurs primarily in the sun-exposed head and neck region of elderly individuals. It was originally described in 1963 and is generally considered to be within the same spectrum as pleomorphic dermal sarcoma, with the latter entity showing deeper invasion into the subcutaneous and deeper tissues and/or adverse features such as necrosis and lymphovascular or perineural invasion. Atypical fibroxanthoma may have a distinct pathogenesis involving ultraviolet light-induced mutations in the p53 gene. Recognition and proper management of this lesion is critical to prevent continued local growth and tissue damage [1-3].

Atypical fibroxanthoma exhibits an array of histologic findings, but classically contains pleomorphic cells arranged in a spindle pattern mixed with atypical histiocyte-like (epithelioid) cells, resembling the findings of pleomorphic sarcoma. Histologically, atypical fibroxanthoma is a high-grade pleomorphic neoplasm, although it behaves as a relatively low-aggression cutaneous malignancy when appropriately excised. Indeed, despite alarming high-grade histologic features, atypical fibroxanthoma carries a favorable prognosis. Surgical excision, including the use of the Mohs micrographic technique, is generally curative and has a recurrence rate of less than 5% [3].

The histologic variant of atypical fibroxanthoma with osteoclast-like cells is rare, with only 18 previously reported cases in the literature. Osteoclast-like giant cells are large, non-neoplastic, multinucleated giant cells that resemble bone-resorbing osteoclasts; they may appear in the stroma of various benign and malignant tumors. We report a 79-year-old woman with an atypical fibroxanthoma with numerous osteoclast-like giant cells that presented as an eyebrow and upper eyelid tumor. Immunohistochemical staining of the biopsy specimen supported the diagnosis. It was successfully extirpated with Mohs micrographic surgery [4-13].

## Case presentation

A 79-year-old Caucasian woman presented for the evaluation and treatment of a rapidly growing lesion on her right lateral eyebrow and upper eyelid that first appeared approximately one month ago. She had a prior history of three basal cell carcinomas, one squamous cell carcinoma, and one melanoma. Her past medical history was also significant for hypertension controlled with carvedilol, hydrochlorothiazide, and losartan potassium, as well as hypercholesterolemia managed with rosuvastatin. Her medical history was unremarkable for immunosuppression, and she was a former tobacco smoker.

Cutaneous examination of the right lateral eyebrow and adjacent right upper eyelid revealed a 2.0 x 2.0-centimeter crateriform, ulcerated, pink nodule that did not appear to be fixed clinically to the underlying orbital rim (Figure 1). There was no preauricular or cervical lymphadenopathy palpated. She was afebrile, with no evidence of acute distress, and had normal vital signs. The clinical differential diagnosis of the lesion included amelanotic melanoma, angiosarcoma, atypical fibroxanthoma/pleomorphic dermal sarcoma, basal cell carcinoma, Merkel cell carcinoma, and squamous cell carcinoma.

**Figure 1:**
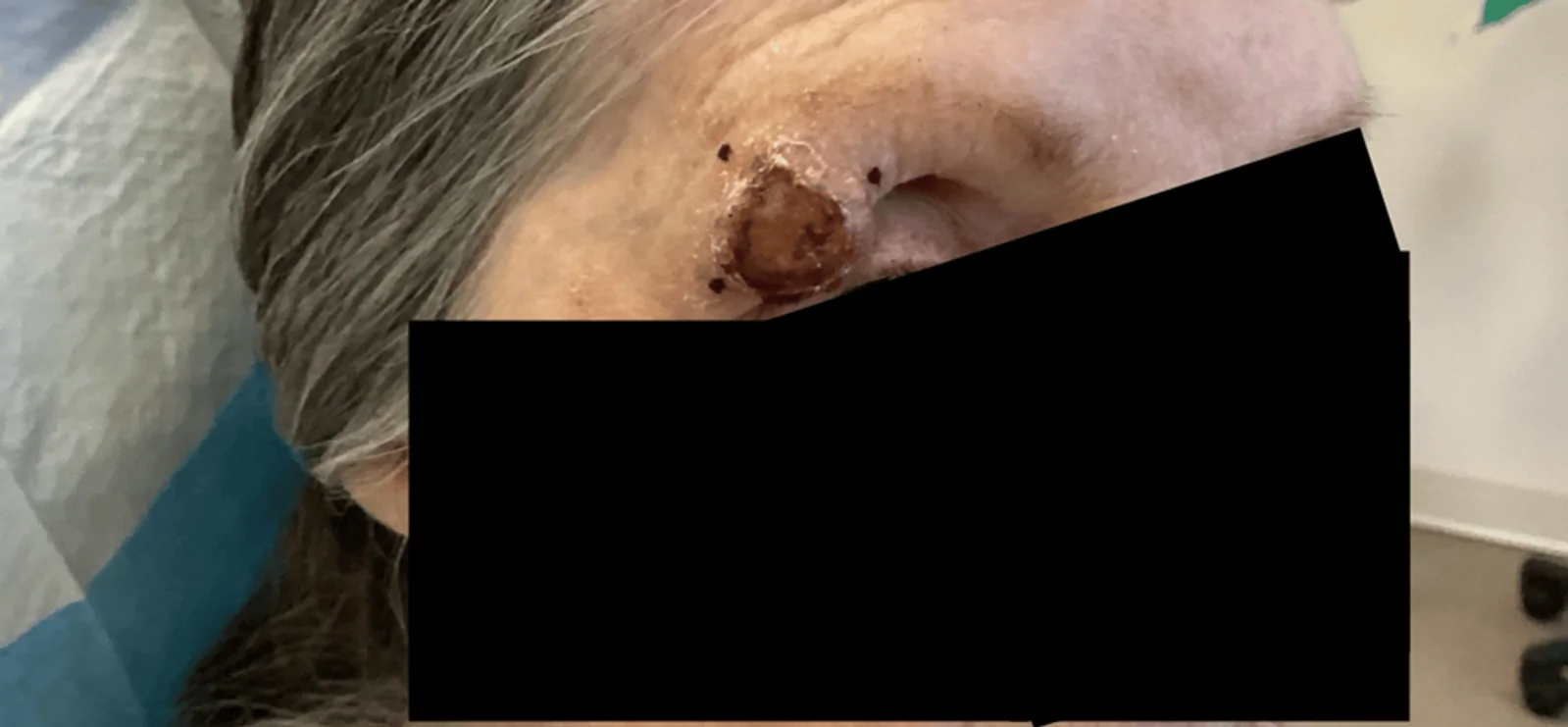
Clinical presentation of atypical fibroxanthoma with osteoclast-like giant cells in a 79-year-old Caucasian woman. A 2.0 x 2.0-centimeter isolated, crateriform, ulcerated nodule is located on the right lateral eyebrow and adjacent upper eyelid region. The tumor is outlined by three purple dots at the 3-, 9-, and 12-o’clock positions.

A shave biopsy of the lesion was performed. Microscopic examination of the tissue specimen revealed an atypical spindle cell neoplasm with numerous osteoclast-like giant cells. The spindle cell component demonstrated hyperchromatic nuclei and occasional mitotic figures (Figures 2-4).

**Figure 2:**
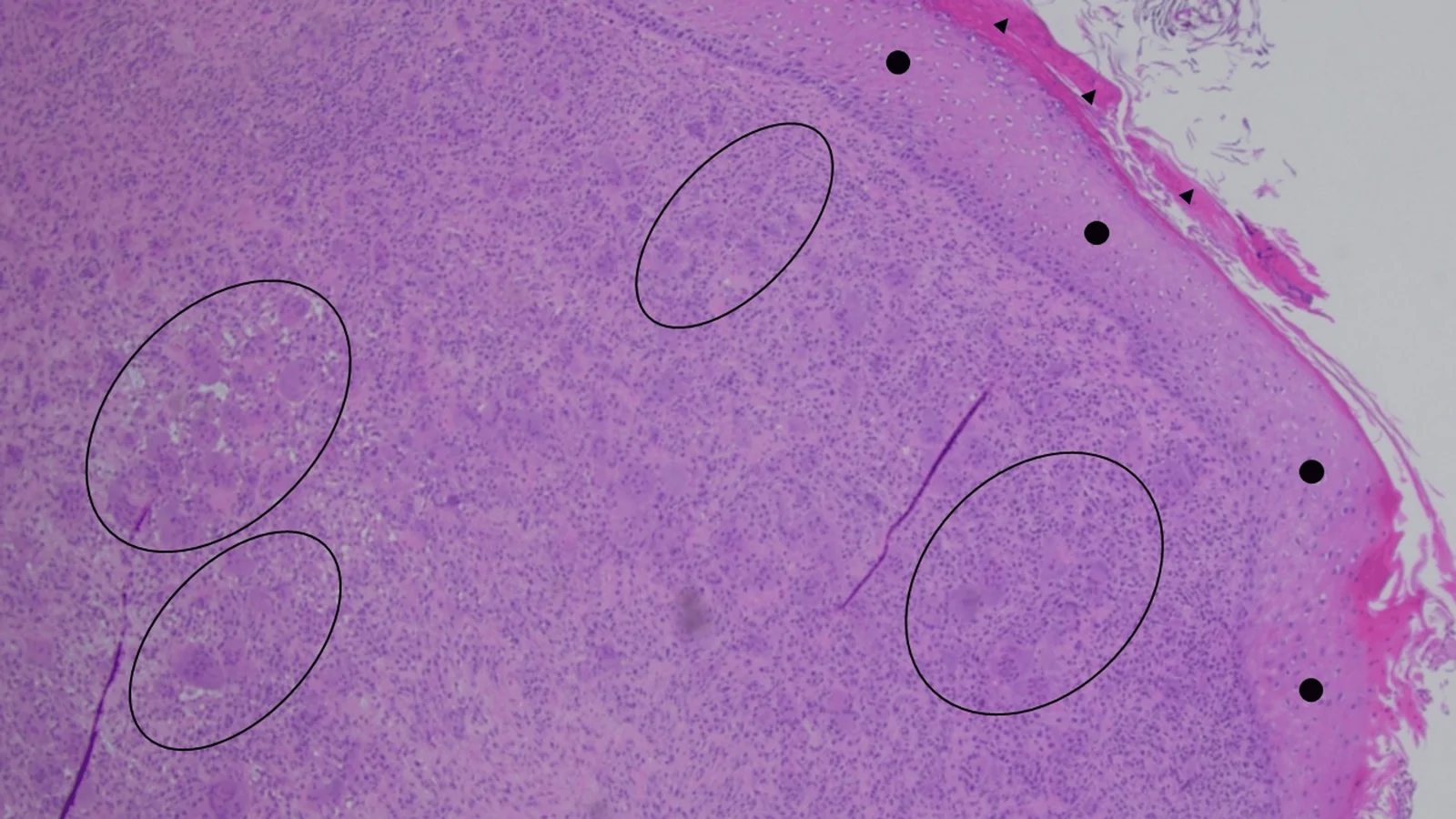
Hematoxylin and eosin-stained section of eyebrow and upper eyelid tumor. Low-magnification view of the tumor demonstrates giant cells in the dermis (black ovals). There is overlying parakeratosis (black arrowheads) and acanthosis of the epidermis (black circles) (hematoxylin and eosin, x10).

**Figure 3:**
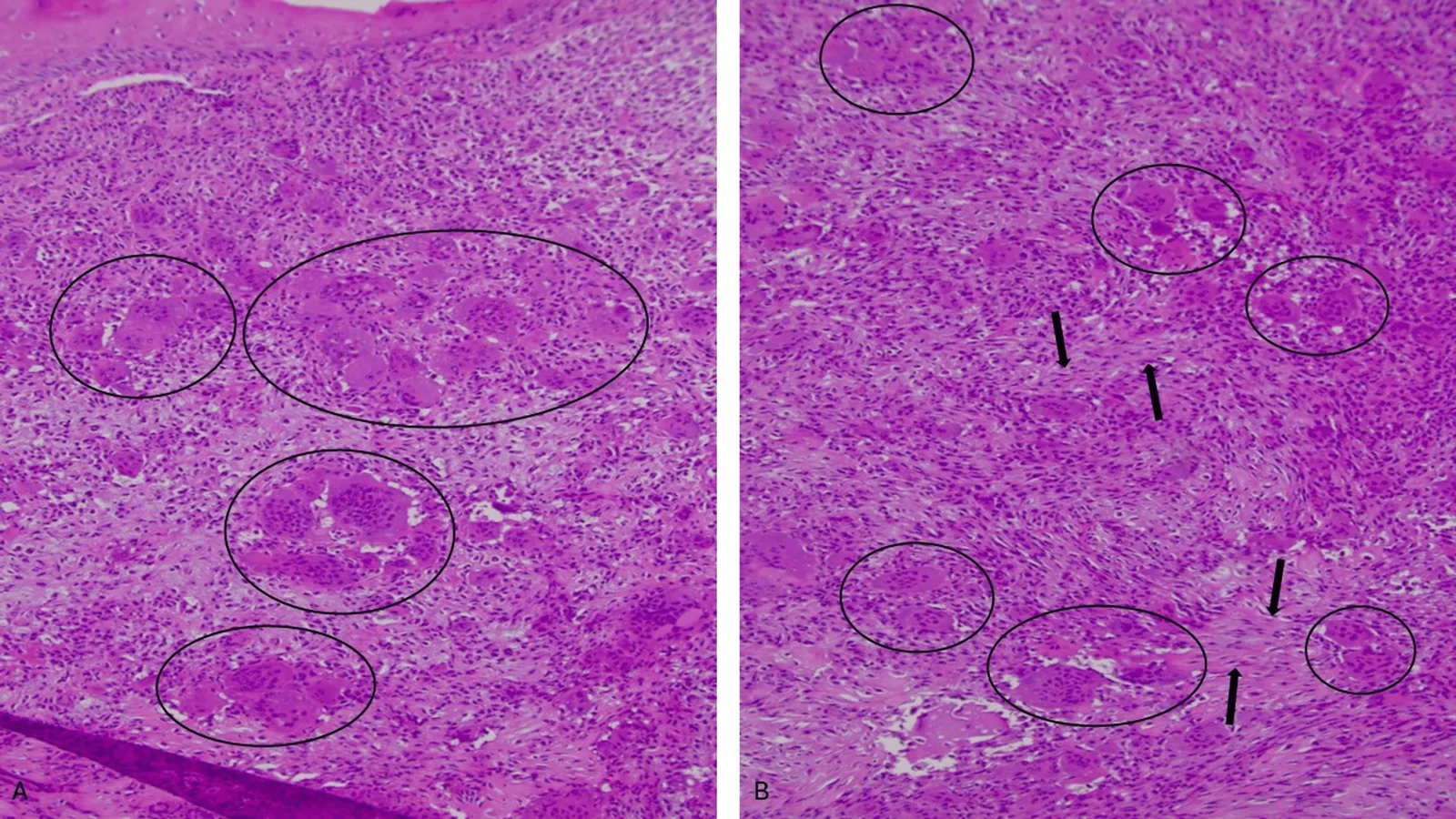
Atypical fibroxanthoma with osteoclast-like giant cells after staining with hematoxylin and eosin. Closer magnification views show multinucleated giant cells (black ovals) in Figure 3A. Black ovals surround some of the giant cells, and black arrows point to spindled tumor cells in Figure 3B (hematoxylin and eosin: A, x40; B, x20).

**Figure 4:**
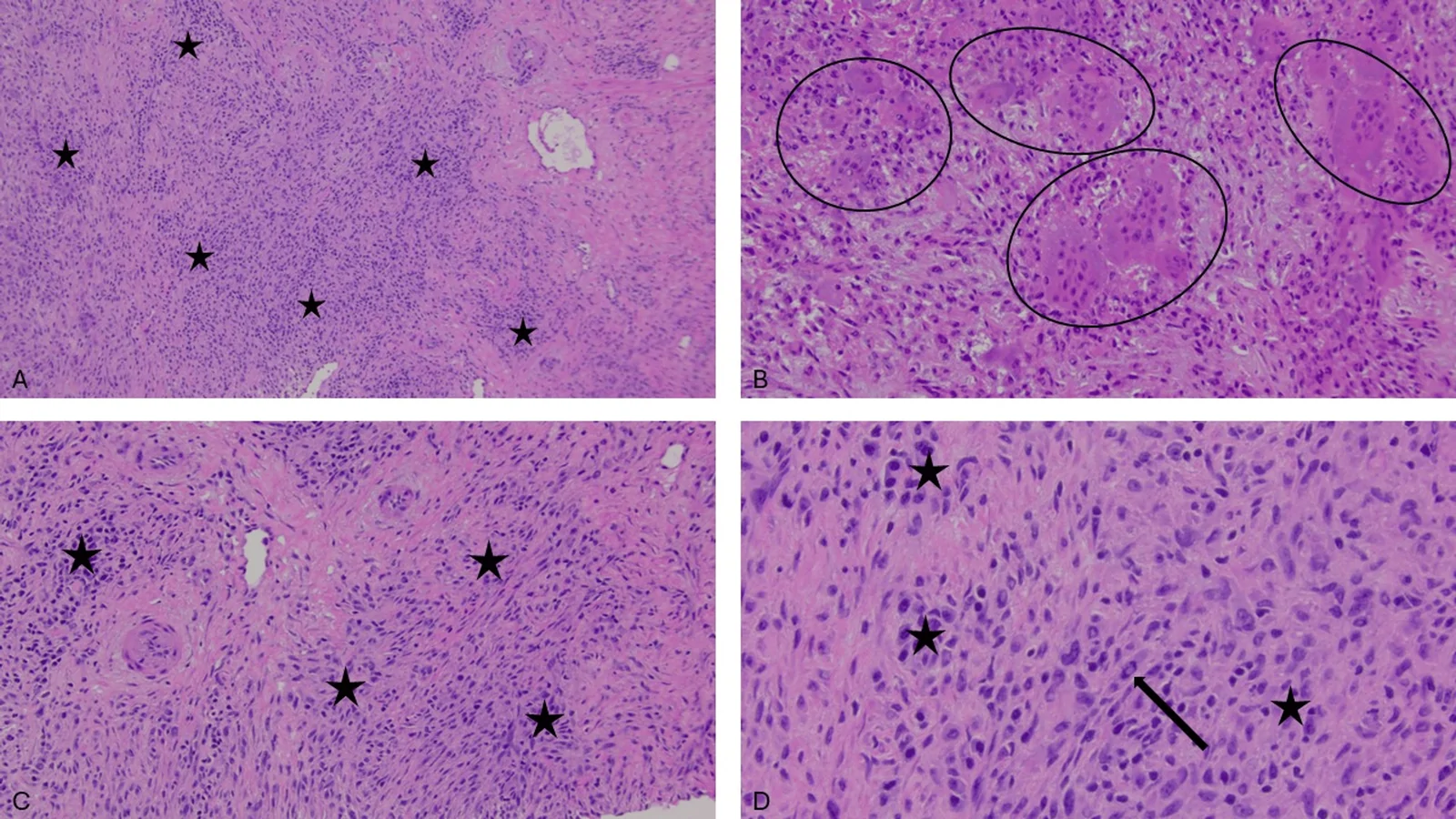
Osteoclast-like giant cells are an uncommon pathologic finding in atypical fibroxanthoma. Black stars indicate epithelioid and spindle tumor cells in Figure 4A. Black ovals encircle osteoclast-like giant cells in Figure 4B. The epithelioid and spindle tumor cells (black stars) are also present in Figures 4C-4D. A black arrow in Figure 4D indicates a mitotic figure within an atypical spindle cell (hematoxylin and eosin: A, x20; B, x40; C, x20; D, x40).

Immunohistochemical staining with S100, SRY-related HMG-box 10 (SOX-10), Preferentially Expressed Antigen in Melanoma (PRAME), cytokeratin 5/6, and cytokeratin AE1/AE3 were negative, excluding a melanocytic and keratinocytic neoplasm (Figure 5). Immunostaining with both cluster of differentiation 10 (CD10) (Figure 6) and cluster of differentiation 68 (CD68) (Figure 7) showed positivity within the malignant spindle cell component of the tumor, while CD68 also stained the multinucleated giant cells. Based on these histologic findings, a diagnosis of atypical fibroxanthoma with a predominance of osteoclast-like giant cells was made.

**Figure 5:**
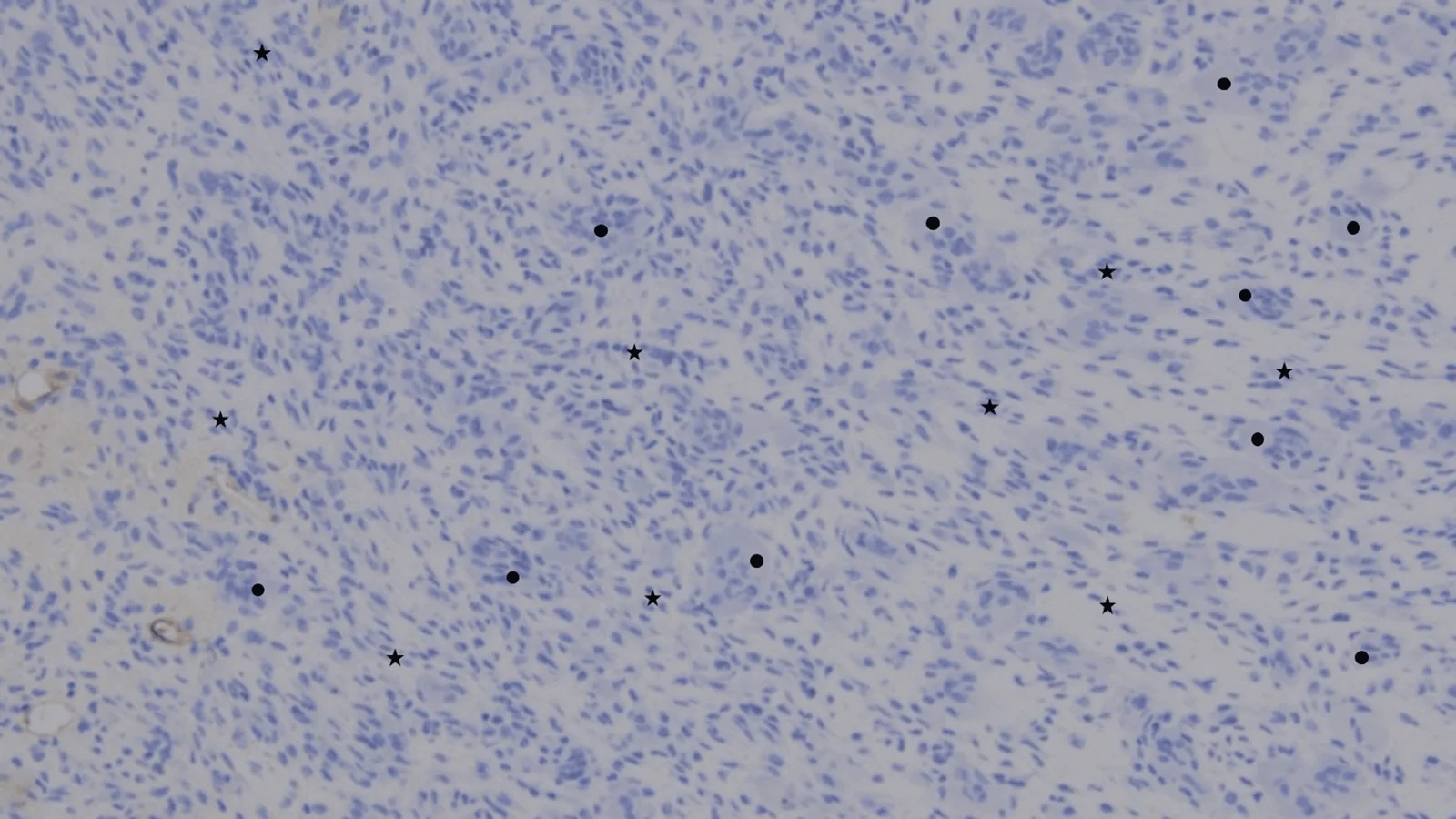
Cytokeratin immunohistochemistry staining of atypical fibroxanthoma with osteoclast-like giant cells. Cytokeratin staining is negative in all components of the neoplasm. Black circles indicate giant cells, and black stars indicate epithelioid and spindle tumor cells (cytokeratin AE1/AE3, x40).

**Figure 6:**
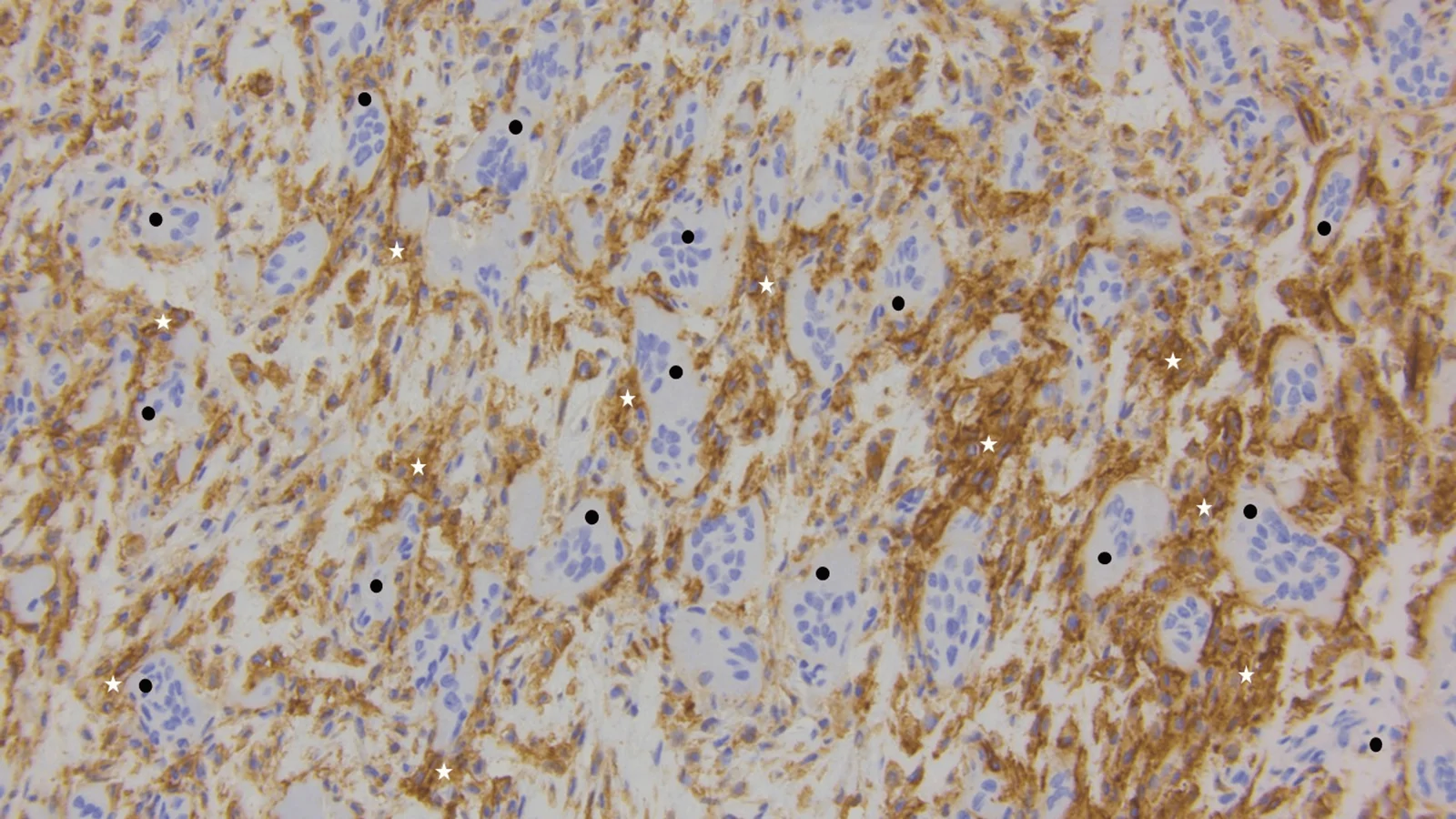
Cluster of differentiation 10 (CD10) immunohistochemistry staining of atypical fibroxanthoma with osteoclast-like giant cells. The CD10 staining is positive within the malignant spindle cell component of the neoplasm. CD10 staining of atypical fibroxanthoma with osteoclast-like giant cells demonstrates positive staining only within the tumor cells. White stars indicate spindle and epithelioid tumor cells, and black circles denote multinucleated giant cells (CD10, x 40).

**Figure 7:**
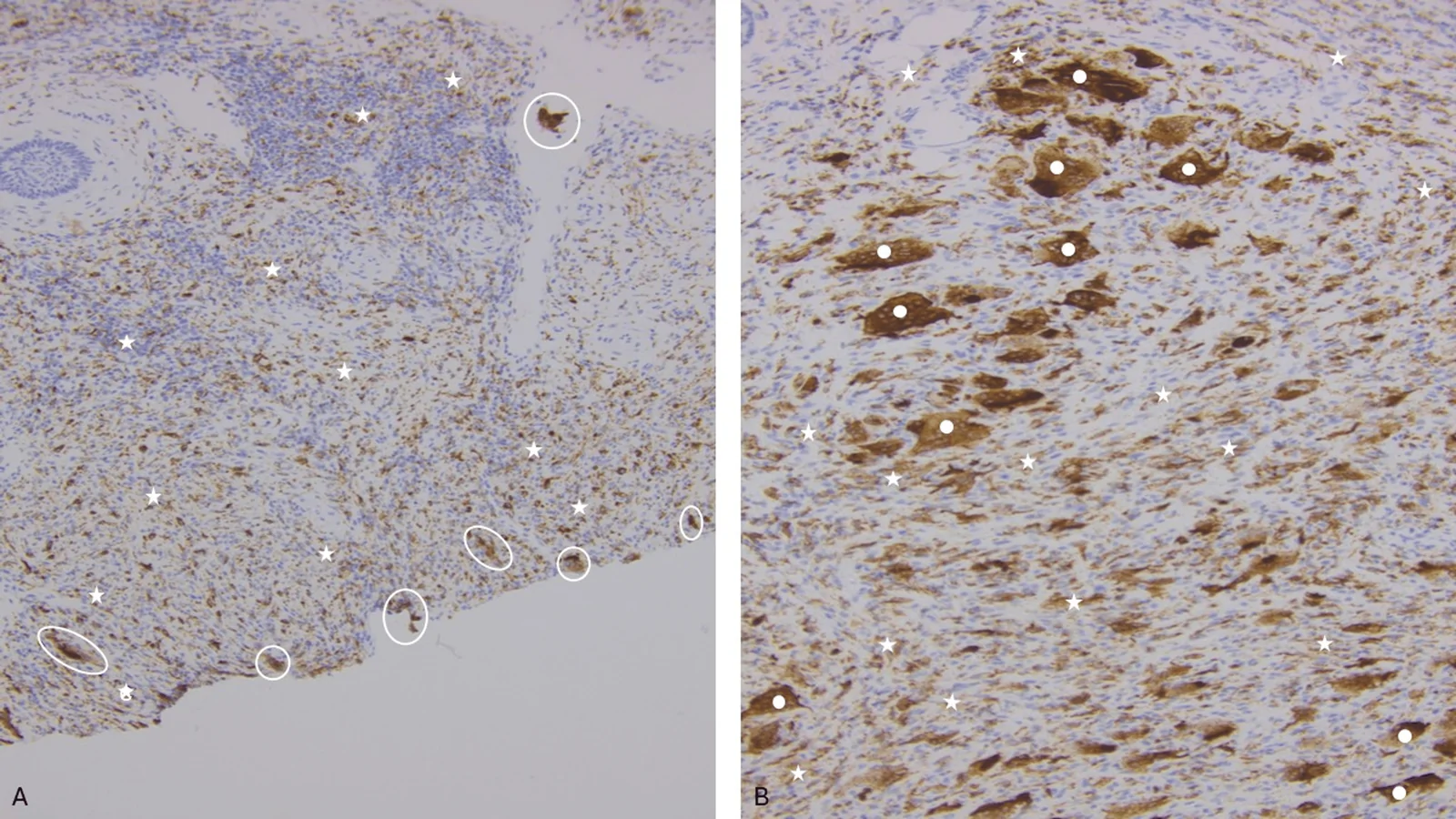
Cluster of differentiation 68 (CD68) immunohistochemistry staining of atypical fibroxanthoma with osteoclast-like giant cells. CD68 staining shows patchy positivity within the spindle cell component (Figure 7A) as well as in the osteoclast-like giant cells (Figure 7B). White stars denote spindle and epithelioid tumor cells, and white ovals indicate giant cells in Figure 7A. White stars denote the spindle and epithelioid tumor cells, and the white circles indicate the giant cells in Figure 7B (CD68: A, x10; B, x20).

The patient underwent Mohs micrographic surgery for treatment due to the anatomic location and size of the lesion. Complete clearance of the atypical fibroxanthoma with histologically negative margins was achieved after the first stage of Mohs surgery. The resulting defect measured 2.4 x 2.3 centimeters and extended to a depth of superficial muscular fascia (Figure 8).

**Figure 8:**
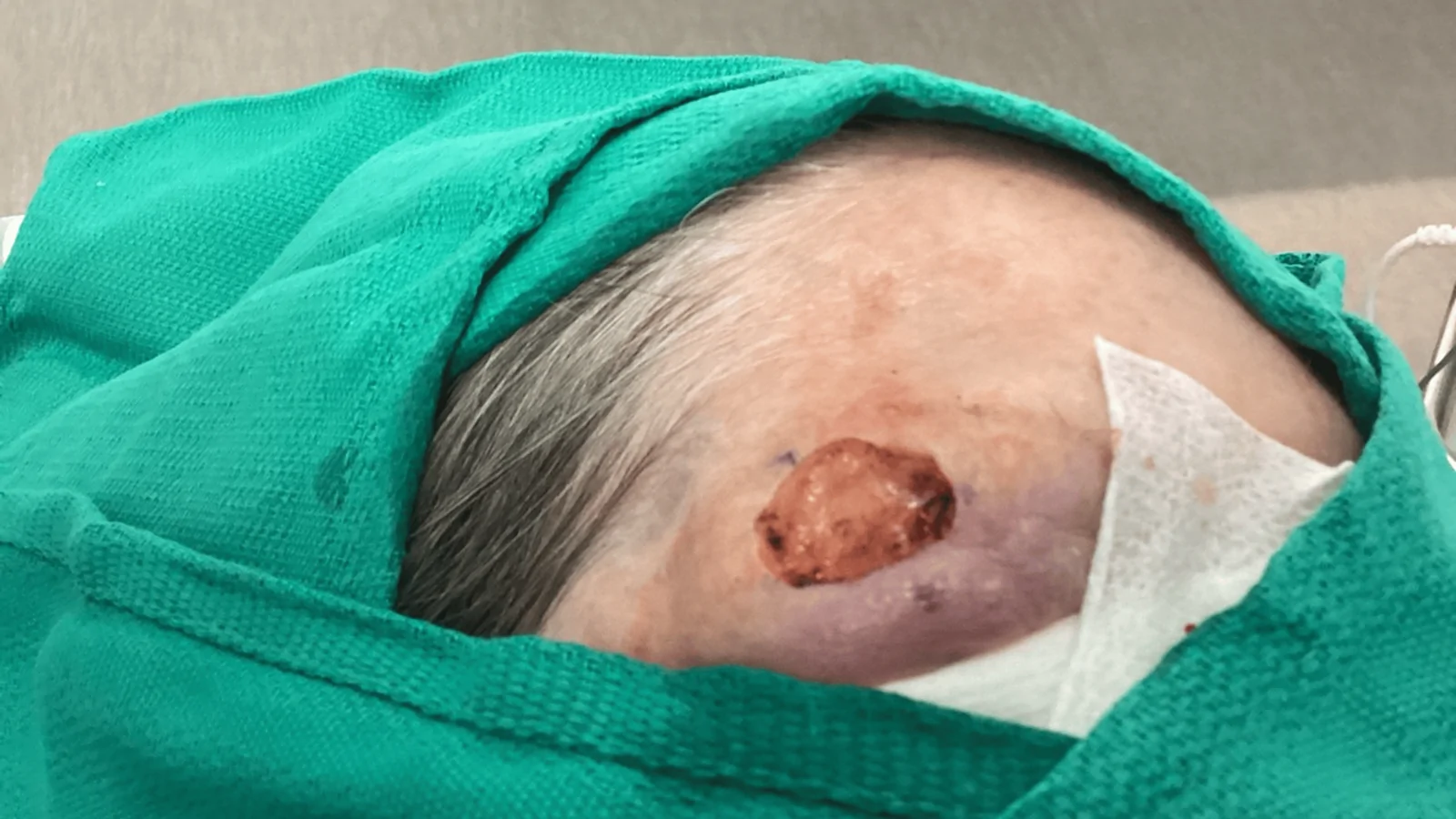
Removal of the right eyebrow and upper eyelid tumor was performed. The surgical wound following complete margin clearance with one stage of Mohs micrographic surgery resulted in a 2.3 x 2.4-centimeter defect that extended to a depth of the superficial muscular fascia. The Mohs defect is outlined by three purple dots at the 12-, 3-, and 6-o’clock positions.

The surgical wound was reconstructed via an advancement flap utilizing tissue reservoirs lateral and medial to the defect (Figure 9). Her postoperative course was uneventful. There was no evidence of motor nerve impairment or recurrent lesion, and the patient had an acceptable cosmetic result at her one-month follow-up visit (Figure 10).

**Figure 9:**
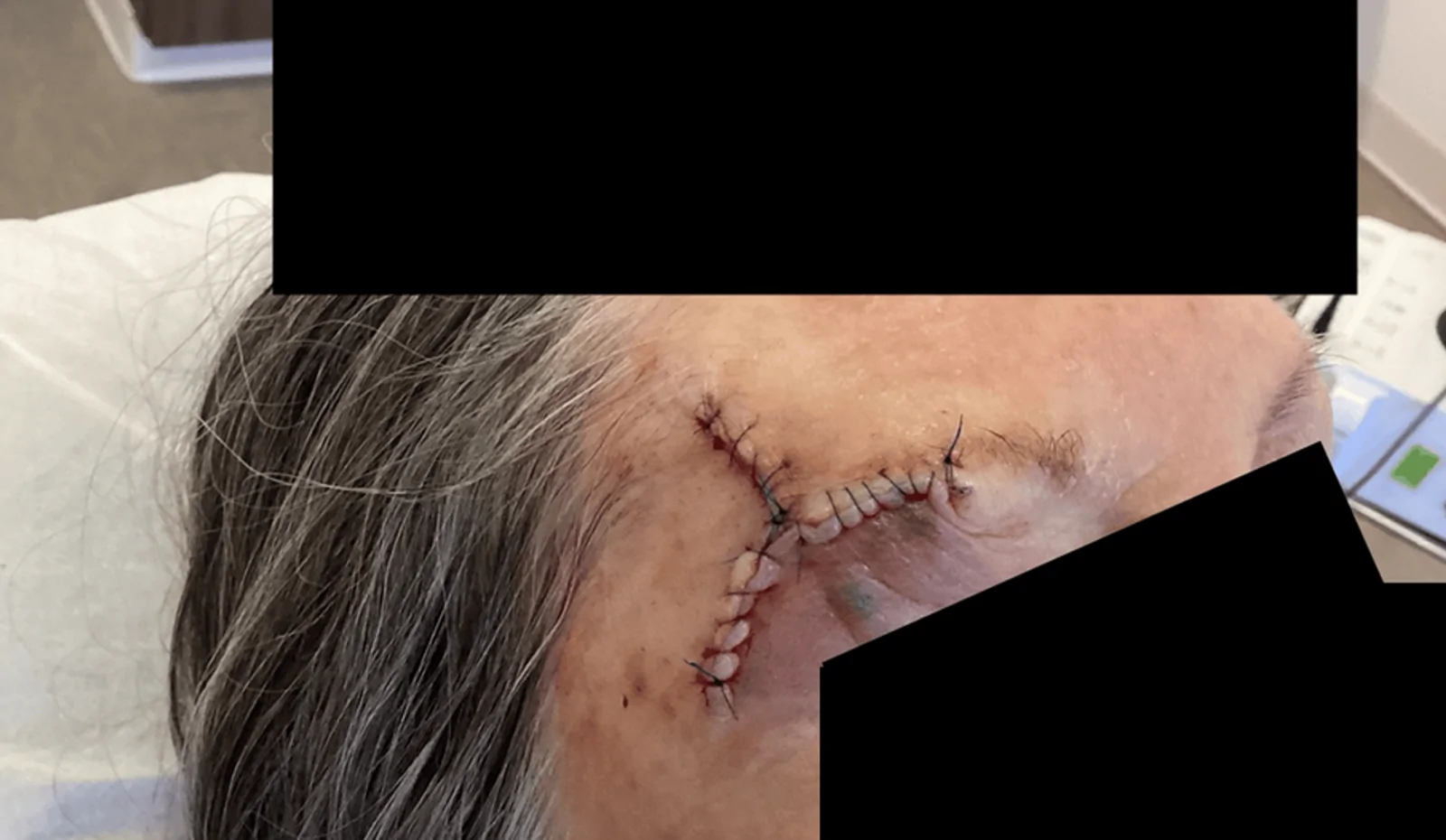
Reconstruction of the surgical wound after tumor extirpation. The immediate postoperative result following repair with a bilateral advancement flap is shown. Tissue reservoirs medial and lateral to the defect were elevated and advanced, with attention given to preserving eyebrow symmetry.

**Figure 10:**
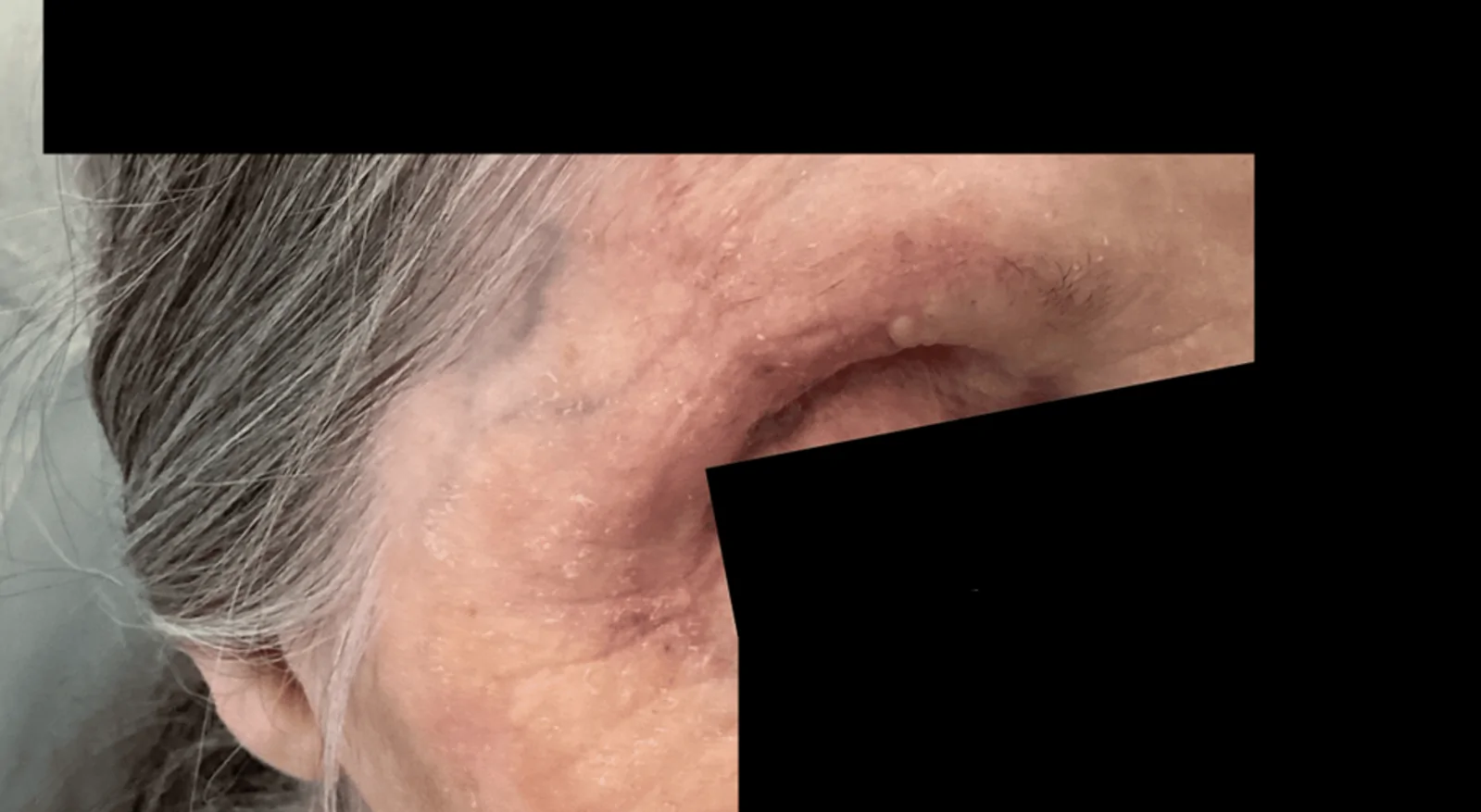
Postoperative follow-up at one month. There is excellent healing of the surgical site following surgery. In addition, there is no clinical evidence of recurrent malignancy. An acceptable functional and aesthetic result can be observed.

## Discussion

Atypical fibroxanthoma is an uncommon, primary, dermal-based tumor that principally arises on the sun-exposed skin in the head and neck region of elderly individuals. In contrast to its strikingly atypical histology, atypical fibroxanthoma behaves in an indolent fashion, with 10-year and 20-year disease-specific survival rates after treatment of 100% and 98%, respectively. Surgical removal of atypical fibroxanthoma is the standard approach to its management. When available, Mohs micrographic surgery is the preferred technique with a reported post-operative recurrence rate of 3.4%. Alternatively, wide local excision with a one-centimeter margin of normal-appearing skin, followed by pathologic margin evaluation, has also been performed for atypical fibroxanthoma [1,2,5,14].

Clinically, atypical fibroxanthoma typically presents as a one-to-two-centimeter, solitary and well-circumscribed, red or pink nodule that may have ulceration, crust, or scale. The differential diagnosis of atypical fibroxanthoma usually includes amelanotic melanoma, basal cell carcinoma, leiomyosarcoma, Merkel cell carcinoma, squamous cell carcinoma, and pleomorphic dermal sarcoma [1,2,14].

The pathophysiology of atypical fibroxanthoma likely involves ultraviolet-induced pyrimidine dimer formation as well as mutations in the tumor suppressor gene p53. Various rare genetic disorders have correlations with p53-induced atypical fibroxanthoma formation, including Li-Fraumeni syndrome (with germline p53 mutations) and xeroderma pigmentosa. Studies have documented mutations in the H-Ras and K-Ras genes in pleomorphic dermal sarcoma, a more aggressive entity. These mutations, however, have not been noted in atypical fibroxanthoma [1,3].

The diagnosis of atypical fibroxanthoma can be confirmed by histopathological examination of a skin biopsy specimen. Although atypical fibroxanthoma may present with a variety of histopathologic subtypes, it classically demonstrates a dermal-based neoplasm with pleomorphic spindle and/or epithelioid cells. Mitoses, including some atypical mitoses, are often present. Atypical fibroxanthoma may also have a variety of additional features, including clear cell change, granular cell change, hemorrhagic and pseudoangiomatous areas, keloid-like areas, myxoid features, and osteoclast-like giant cells [13].

Atypical fibroxanthoma with osteoclast-like giant cells is a rare entity. Including the patient in this report, only 19 patients have had this histopathologic variant since its first report in 1988. The clinical features of individuals with atypical fibroxanthoma with osteoclast-like giant cells are summarized in Table 1 [4-13]. The histopathologic and immunohistochemical features are summarized in Table 2 [4-13].

**Table 1:** Summary of clinical features of atypical fibroxanthoma with osteoclast-like giant cells. C: case; CR: current report; F: female; G: gender; L: left; M: male; mm: millimeter; NR: not reported; R: right; Ref: reference; Y: years ^a ^In one of the three patients, the atypical fibroxanthoma originated from the skin of a finger. The tumor site was not described for the other patients in the report. ^b ^A retrospective study of 171 atypical fibroxanthomas in 169 patients (two patients had two tumors each). There were 128 men and 41 women; the male-to-female ratio was 3:1. The patients ranged from 41 to 97 years; the median age was 74 years, and the mean age was 75 years. Only 1.2% of the atypical fibroxanthomas (two tumors) had osteoclast-like giant cells. Their smaller nuclei, monomorphism, and lack of atypia or mitotic activity distinguished them from neoplastic cells. The investigators interpreted them as fused aggregates of reactive macrophages [12]. ^c ^A retrospective study of 66 atypical fibroxanthomas in 59 men and seven women ranging from 55 to 95 years (mean, 77 years). Osteoclast-like giant cells were noted in four tumors (6.1%). The researchers postulate that the atypical "intermediate" giant cells within the atypical fibroxanthoma represent the extreme step of large pleomorphic multinucleated giant cells rather than a transformation, a fusion, or both of reactive histiocytes [13].

C	Age (y)	G	Site	Clinical presentation	Authors	Ref
1	71	F	Digit^a^	15-30 mm rapidly growing ulcerated nodules	Orlandi et al.	[6] C1
2	74	F	R cheek	13 mm firm, hyperemic, ulcerated nodule	Wilson et al.	[9]
3	79	F	R lateral eyebrow area	20 mm crateriform, ulcerated pink nodule	Martinelli et al.	CR
4	87	F	Forehead	8 mm subcutaneous nodule, recurrent after excision as 11 mm yellow subcutaneous nodule	Val-Bernal et al.	[10]
5	88	F	R mandible	15 mm pink-purple dome-shaped nodule with central crust	Anderson et al.	[5]
6	65	M	L forearm	10 mm nodule	Tomaszewski and Lupton	[8] C3
7	69	M	L neck	10 mm nodule	Tomaszewski and Lupton	[8] C2
8	73	M	R temple	10 mm pearly red polypoid nodule	Khan and Cockerell	[7]
9	77	M	Digit^a^	15-30 mm rapidly growing ulcerated nodules	Orlandi et al.	[6] C2
10	77	M	R temple	10 mm nodule	Tomaszewski and Lupton	[8] C1
11	77	M	R central parietal scalp	Enlarging red nodule	Shaker et al.	[4]
12	80	M	Digit^a^	15-30 mm rapidly growing ulcerated nodules	Orlandi et al.	[6] C3
13	90	M	L zygoma	13 mm ulcerated nodule	Ferrara et al.	[11]
14-15^b^	NR	NR	NR	NR	Beer et al.	[12]
16-19^c^	NR	NR	NR	NR	Luzar and Calonje	[13]

**Table 2:** Summary of histopathologic and immunohistochemical features of atypical fibroxanthoma with osteoclast-like giant cells. C: case; CD: cluster of differentiation; CK: cytokeratin; CR: current report; HMB45: human melanoma black 45; HPF: high-powered fields; IHC: immunohistochemistry; Ki: Kiel; MART-1: melanoma antigen recognized by T cells 1; Melan-A: melanocyte antigen; MNGC: multi-nucleated giant cells; NR: not reported; PRAME: preferentially expressed antigen in melanoma; RANK: receptor activator of nuclear factor-kappa B; Ref: reference; SMA: smooth muscle actin; SOX-10: SRY-related HMG-box 10; ⍺: alpha ^a ^A retrospective study of 171 atypical fibroxanthomas in 169 patients (two patients had two tumors each). There were 128 men and 41 women; the male-to-female ratio was 3:1. The patients ranged from 41 to 97 years; the median age was 74 years, and the mean age was 75 years. Only 1.2% of the atypical fibroxanthomas (two tumors) had osteoclast-like giant cells. Their smaller nuclei, monomorphism, and lack of atypia or mitotic activity distinguished them from neoplastic cells. The investigators interpreted them as fused aggregates of reactive macrophages [12]. ^b ^A retrospective study of 66 atypical fibroxanthomas in 59 men and seven women ranging from 55 to 95 years (mean, 77 years). Osteoclast-like giant cells were noted in four tumors (6.1%). The researchers postulate that the atypical "intermediate" giant cells within the atypical fibroxanthoma represent the extreme step of large pleomorphic multinucleated giant cells rather than a transformation, a fusion, or both of reactive histiocytes [13].

C	Histologic features	IHC positive	IHC negative	Authors	Ref
1	MNGC and osteoclast-like giant cells, atypical spindle cells among histiocyte-like cells	Anti-RANK	CD68, ⍺-SMA	Orlandi et al.	[6] C1
2	Haphazardly arranged spindle-shaped and multinucleated polygonal cells, peripherally arranged osteoclast-like giant cells	Vimentin, ⍺-1 antitrypsin, lysozyme	S100, CK	Wilson et al.	[9]
3	Atypical spindle cell neoplasm with numerous osteoclast-like giant cells, mitotic figures	CD68, CD10	CK, S100, PRAME, SOX-10	Martinelli et al.	CR
4	Osteoclast-like giant cells, atypical monocytic cells, numerous mitoses	CD68, ⍺-1 antitrypsin, ⍺-1 antichy-motrypsin	S100, HMB45, myoglobin, desmin	Val-Bernal et al.	[10]
5	Spindle oval and osteoclast-like giant cells with pleomorphism and five atypical mitoses per 10 HPF	CD68, CD10, Ki-67	p63, CK, desmin, S100, Mart-1, Melan-A, CD31, CD34	Anderson et al.	[5]
6	Atypical spindle cells with mitoses, bizarre tumor giant cells with osteoclast-like giant cells	Vimentin, Factor XIIIa, lysozyme, ⍺-1 antitrypsin, specific muscle actin, KP-1	S100, HMB45, desmin	Tomaszewski and Lupton	[8] C3
7	Atypical spindle cells with mitoses, bizarre tumor giant cells with osteoclast-like giant cells	Vimentin, Factor XIIIa, lysozyme, ⍺-1 antitrypsin, specific muscle actin, KP-1	S100, HMB45, desmin	Tomaszewski and Lupton	[8] C2
8	MNGC, histiocytic cells, atypical, histiocytic cells	CD68, MAC 387, ⍺-1 antichymo-trypsin	S100, HMB45, CK	Khan and Cockerell	[7]
9	MNGC and osteoclast-like giant cells, atypical spindle cells among histiocyte-like cells	Anti-RANK	CD68, ⍺-SMA	Orlandi et al.	[6] C2
10	Atypical spindle cells with mitoses, bizarre tumor giant cells with osteoclast-like giant cells	Vimentin, Factor XIIIa, lysozyme, ⍺-1 antitrypsin, specific muscle actin, KP-1	S100, HMB45, desmin	Tomaszewski and Lupton	[8] C1
11	Dermal-based spindle cell neoplasm with many osteoclast-like MNGC	CD68, SMA, CD10	CK, S100, desmin, SOX-10	Shaker et al.	[4]
12	MNGC and osteoclast-like giant cells, atypical spindle cells among histiocyte-like cells	Anti-RANK	CD68, ⍺-SMA	Orlandi et al.	[6] C3
13	Uniformly distributed osteoclast-like giant cells with 10-30 nuclei, pleomorphic histiocytic cells, mitotic figures	SMA, lysozyme, S100 (in giant cells)	CD68, keratin (CAM5.2)	Ferrara et al.	[11]
14-15^a^	NR	CD68	Actin, Melan-A, HMB45	Beer et al.	[12]
16-19^b^	NR	NR	NR	Luzar and Calonje	[13]

Epidemiologic individualized clinical information was provided for 13 of the 19 patients with atypical fibroxanthoma presenting with osteoclast-like giant cells. There were eight men and five women. Therefore, there was nearly a twofold increase in prevalence in men compared to women.

The patients ranged in age from 71 to 90 years (median, 77 years). The men ranged from 65 to 90 years (median, 77 years). The women ranged from 71 to 88 years (median, 79 years) [4-13]. Nearly all cases occurred in the sun-exposed head and neck region [4-13]. One man’s tumor occurred on his left forearm [8]. One individual’s atypical fibroxanthoma originated from the skin of their finger [6].

The immunohistochemistry observed in atypical fibroxanthoma with osteoclast-like giant cells was variable. Care should be exercised when relying entirely on these stains to diagnose an atypical fibroxanthoma with osteoclast-like giant cells. However, all cases of atypical fibroxanthoma showed CD-10 positive immunoexpression [1,4,5,11,12].

Melanocytic and keratinocyte stains are generally negative in atypical fibroxanthomas. Classic atypical fibroxanthoma stains negatively for S100, but the variant with osteoclast-like giant cells can demonstrate positive S100 expression. Smooth muscle actin and CD68 staining can be variable [1,4,5,11,12].

The origin of the osteoclast-like cells remains unknown. One hypothesis suggests that the giant cells may represent multinucleated histiocytes. Other investigators, based on reactive immunostaining characteristics, believe these cells represent differentiation of neoplastic cells rather than a transformation or fusion of normal histiocytes. Further research is required to elucidate the definitive origin of the osteoclast-like giant cells seen in atypical fibroxanthoma [6,8].

It is important to consider the histopathologic mimics of this rare lesion to avoid diagnostic pitfalls. Sarcomatoid squamous cell carcinoma has been reported with osteoclast-like giant cells. Positive cytokeratin staining may assist in differentiating poorly differentiated squamous cell carcinomas; atypical fibroxanthoma usually does not demonstrate staining with cytokeratin [12,15-20].

Additionally, melanoma must be ruled out. Balloon cell, clear cell, or spindle cell melanoma may resemble atypical fibroxanthoma on pathologic examination. Melanoma with osteoclast-like giant cells has also been reported. Positive staining after evaluation with melanoma antigen recognized by T cells 1 (MART-1) and human melanoma black 45 (HMB-45) would suggest melanoma [12,15-20].

Leiomyosarcoma can also resemble atypical fibroxanthoma on histopathology. It can also present with osteoclast-like giant cells. Smooth muscle actin may be positive in both leiomyosarcoma and atypical fibroxanthoma. Importantly, desmin staining is typically reactive within leiomyosarcoma only, making its inclusion necessary for accurate diagnosis [12,15-20].

Pleomorphic dermal sarcoma must also be considered in the pathologic differential diagnosis of atypical fibroxanthoma. However, this entity is usually distinguished from atypical fibroxanthoma by at least one of the following more aggressive histologic features: more than minimal subcutaneous involvement, coagulative necrosis, lymphovascular invasion, and perineural invasion [2,3]. Finally, osteoclast-like giant cells may be seen in giant cell tumor of the skin. In addition, osteoclast-like giant cells can also be observed in a variety of benign and reactive cutaneous lesions [12,15-20].

The patient presented in this report demonstrated a typical clinical presentation for atypical fibroxanthoma with osteoclast-like giant cells. Her age of 79 is consistent with the median age of women previously reported in the literature. Additionally, her extensive actinic damage of the head and neck region, as well as her history of prior non-melanoma and melanoma skin cancers, is consistent with atypical fibroxanthoma’s predilection for sun-exposed cutaneous surfaces. While the diagnosis of atypical fibroxanthoma with osteoclast-like giant cells was considered from the features seen on routine hematoxylin and eosin-stained tissue sections, it was confirmed with the addition of immunohistochemical stains. Specifically, negative cytokeratin and melanocytic stains were able to rule out sarcomatoid squamous cell carcinoma and melanoma, respectively, while positive CD10 and CD68 stains both supported a diagnosis of atypical fibroxanthoma [4-13].

## Conclusions

An elderly immunocompetent woman with a biopsy-proven atypical fibroxanthoma with osteoclast-like giant cells on the right eyebrow area has been reported. The clinical differential diagnosis included amelanotic melanoma, angiosarcoma, basal cell carcinoma, Merkel cell carcinoma, and squamous cell carcinoma. In addition to atypical fibroxanthoma, dermal tumors with osteoclast-like giant cells include leiomyosarcoma, melanoma, and sarcomatoid squamous cell carcinoma. Immunohistochemical stains may help to establish a definitive diagnosis. Our patient’s tumor was not only CD10-positive but was also S100 (melanoma stain) and cytokeratin (squamous cell carcinoma stain) negative. The giant cells were also CD68 positive. The lesion was successfully treated with Mohs micrographic surgery. The patient is the 19th reported individual with this uncommon histopathologic presentation of atypical fibroxanthoma in the literature. It is important to consider the diagnosis of atypical fibroxanthoma with osteoclast-like giant cells when evaluating skin tumors in sun-exposed locations, especially the head and neck, that present with osteoclast-like giant cells.
